# Increased production of hydrogen peroxide by peripheral blood monocytes associated with smoking exposure intensity in smokers

**DOI:** 10.1186/1476-9255-9-45

**Published:** 2012-11-21

**Authors:** Suzana E Tanni, Camila R Correa, Aparecida Y Angeleli, Simone A Vale, Liana S Coelho, Irma Godoy

**Affiliations:** 1 Faculdade de Medicina de Botucatu, Disciplina de Pneumologia, Univ Estadual Paulista, UNESP, Botucatu, São Paulo, Brazil; 2 Faculdade de Medicina de Botucatu, Univ Estadual Paulista, UNESP, Departamento de Patologia, Botucatu, São Paulo, Brazil; 3 Departamento de Clínica Médica da Faculdade de Medicina de Botucatu, UNESP, Distrito de Rubião Júnior, Botucatu, SP, 18618-970, Brazil

**Keywords:** Hydrogen peroxide, Smoking, Oxidative stress, Systemic inflammation, Cultured cells

## Abstract

**Background:**

Smoking is known to be associated with oxidative stress; however, it has not been elucidated whether the oxidative response is influenced by the intensity of smoking exposure.

**Objectives:**

Evaluate the effect of smoking exposure on the secretion of hydrogen peroxide (H_2_O_2_) by the peripheral blood monocytes of smokers.

**Methods:**

A total of 25 smokers (50.3±8.8 years, 48% male) underwent the following evaluations: spirometry, pulse oximetry, body composition and total peripheral blood count. Peripheral blood monocyte (PBM) cultures were isolated and maintained, and IL-6 and TNF-α were measured in the plasma and in the supernatants of spontaneous and stimulated cultures. H_2_O_2_ was evaluated in the supernatants of the PBM cultures, and a subset of the PBM culture supernatants was stimulated with phorbol myristate acetate (PMA). We also evaluated 38 healthy controls (49.1±8.2 years, 42% male).

**Results:**

The spontaneous and stimulated monocytes’ secretion of H_2_O_2_ were statistically higher in the smokers than in the healthy controls (p<0.001). The H_2_O_2_ secretions were statistically significant higher after stimulation with PMA in both groups (p<0.001). In the multiple regression analysis, we identified a positive, statistically significant association between pack-years of smoking and the spontaneous secretion of H_2_O_2_ by PBM culture, adjusted for potential confounding variables. The association between PBM culture secretion of H_2_O_2_ and the production of TNF-α and IL-6 was not significant.

**Conclusion:**

We identified a positive association between higher production of H_2_O_2_ in smokers and higher smoking exposure during life. The influence of pack-years smoking may be a key modifiable factor in oxidative stress associated to smoking.

## Introduction

Smoking is a major agent of environmental pollution and is the leading preventable cause of death worldwide [1]. According to data from the World Health Organization, smoking is responsible for 85% of deaths due to chronic obstructive pulmonary disease (COPD), 30% of cancer deaths and 25% of deaths from coronary disease [1]. The development of smoking-related diseases is associated with the deleterious effects of cigarette smoke, including increased oxidative stress and inflammatory processes [2,3].

Oxidative stress is associated with higher concentrations of reactive oxygen specimens, including hydrogen peroxide (H_2_O_2_), and this phenomenon is a characteristic response of inflammatory cells to smoking [4-7]. Oxidative stress can further stimulate the inflammatory process in the airways, increasing the levels of cytokines and the accumulation of polymorphonuclear leukocytes (PMNs) [2,8-10]. Moreover, this stress may induce the oxidation of lipids, lipoproteins, DNA and other proteins and molecules in ways that impair normal cellular function and enable the development of many diseases, such as COPD and atherosclerosis. COPD patients present higher concentrations of H_2_O_2_ in exhaled breath condensate compared with healthy controls [11], which might be associated with smoking exposure. Oxidative stress activates proteins that are associated with the destruction of parenchymal tissue [11].

An association between active smoking and elevated oxidative stress in alveolar macrophages is well described in the literature [2,12,13]; however, whether the intensity of smoking exposure has an impact on oxidative stress has been less thoroughly investigated. In the study of van Beurden et al. [14], the production of superoxide by PMNs was not associated with the intensity of smoking exposure evaluated by smoking history duration. Similarly, Yeh et al. [15] did not find an association with smoking history duration when protein carbonylation was used as a biomarker for oxidative stress. Therefore, the aim of this study was to assess the relationship between systemic oxidative stress, represented by the secretion of H_2_O_2_ by peripheral monocytes, and pack-years of smoking.

## Methods

We evaluated 25 active smokers (52% female) of at least 10 pack-years and 38 healthy controls self-identified as never smokers (57.9% female). All of the participants underwent routine clinical assessments, including spirometry and chest X-ray. The height and weight were measured, and the body mass index (BMI) was calculated (kg/m^2^). The exclusion criteria included pharmacological treatments in the last three months before enrollment in the study, a forced expiratory volume in the first second (FEV_1_)/Forced vital capacity (FVC) ratio < 0.70 or significant reversibility (increase >200 mL or >11% in the value of FEV_1_ expressed as percentage of the predicted value) 20 minutes after the inhalation of a β2-agonist (400 mcg fenoterol) in the spirometry test, diagnosis of any chronic disease, and inability to understand the study protocol.

The Research Ethics Committee of the Botucatu Medical School approved the study design, and all of the participants gave written informed consent.

### Spirometry test and pulse oximetry

The FEV_1_ and FVC were obtained from the flow-volume curve using a spirometer (Ferraris KOKO, Louisville, CO, USA) before and 20 minutes after the inhalation of a β2-agonist (400 mcg fenoterol) according to Brazilian guidelines [16]. The highest value of at least three measurements was selected and expressed as a percentage of reference values [16]. The pulse oximetry (SpO_2_%) was assessed by a portable oximeter (Nonin Medical, Plymouth, MN, USA).

### Peripheral blood monocyte isolation and culture (PBM)

PBM cultures were isolated from heparinized blood drawn between 07:00 – 08:00 using a density gradient followed by plastic adherence, as described elsewhere [17]. In brief, the PBM cultures were isolated from heparinized blood using Histopaque 1077 (Sigma Chemical Co., St Louis, MO, USA) and washed three times with Hanks’ solution (Sigma Chemical Co.). A cell suspension was prepared with PBMCs at 2×10^6^ cells·mL^-1^ in Roswell Park Memorial Institute (RPMI)-1640 medium (Sigma Chemical Co.) supplemented with 5% heat-inactivated autologous serum, 2 mM glutamine, 100 U·mL^-1^ penicillin and 10 mM HEPES (N-2-hydroxyethylpiperazine-N-2-ethanesulfonic acid) buffer (that hereafter will be referred to as mRPMI) and incubated for 2 hours in a humidified atmosphere with 5% CO_2_ at 37°C. Nonadherent cells were removed by three washes with plain RPMI and counted. The adherent cells were provided with fresh mRPMI at a volume of 1 mL·1×10^6^ cells^-1^ and incubated for 24 hours. The supernatants were collected, centrifuged and stored at −80°C until cytokine analysis. After the supernatants were harvested, the adherent cells were stained with nonspecific esterase staining (Sigma Chemical Co.), and the cell viability was determined using a trypan blue exclusion test. The adherent cells contained > 95% monocytes; >98% were viable.

### Interleukin (IL)-6 and tumor necrosis factor alpha (TNF-Î±) measurements

Fasting peripheral blood was collected in the early morning (07.00-08.00 hours), and the plasma was stored at −80°C until analysis. TNF-α and IL-6 in the spontaneous PBM culture supernatant and plasma were assessed in duplicate by highly sensitive commercial kits using the enzyme-linked immunosorbent (ELISA) technique according to the manufacturer’s instructions (BioSource International Inc, CA, USA). The lower detection limits were 0.5 pg/mL for TNF-α and 0.16 pg/mL for IL-6.

### H_2_O_2_ production

The production of H_2_O_2_ was determined according to the method described by Pick & Keisari [18], adapted by Pick & Mizel [19] and also previous published [3]. After an 18-hour incubation period, supernatants of cultures of monocytes were discarded, and the cells were resuspended to the original volume in phenol red solution containing 140 mM of NaCl; 10 mM of phosphate-buffered saline (pH 7); 5.5 mM of dextrose; 0.56 mM phenol red; and 0.01 mg/ml of horseradish peroxidase, type II (Sigma Chemical Co, USA). The supernatants were divided into two parts, incubated with or without 1 μg of phorbol myristate acetate (PMA) and used as positive controls for H_2_O_2_. After 1 hour, the reaction was interrupted by the addition of 0.01 ml of 1 N NaOH. The samples were assayed in quadruplicate. The absorbance was determined using an ELISA reader at 620 nm against a blank containing phenol red and 1 N NaOH.

The results were expressed as nanomoles of H_2_O_2_/2×10^5^ cells using the standard curve established in each assay and made up of known molar concentrations of H_2_O_2_ in phenol red buffer solution. In these experimental conditions, the curve was generated based on the following concentrations: 0.5; 1.0; 1.5 and 2.0 nM of H_2_O_2_.

### Statistical analysis

The results are presented as the means ± SD for normally distributed variables and the median (range) when not normally distributed. The independent t test or Mann–Whitney was used to compare groups according to smoking status. The Chi-square test was used to compare proportions between groups. To explore the association of H_2_O_2_ with markers of systemic inflammation (TNF-α, IL-6 and neutrophils) and with pack-years of smoking, we performed multiple regression analysis with robust standard errors with and without adjustment for potential confounding variables. A p value of less than 0.05 denotes statistically significant difference (“STATA” 10.0 - Corp, College Station, TX, USA).

## Results

The demographic characteristics and the data related to the spirometry, pulse oximetry and BMI of the smokers and controls are shown in Table 1. The smokers exhibited lower values of FEV_1_ (L), FEV_1_/FVC (%) and BMI (kg/m^2^) compared with the healthy controls.

**Table 1:** Demographic characteristics

	**Smokers**	**Controls**	**p value**
	**N=25**	**N=38**	
Age (years)	50.3±8.8	49.1± 8.2	0.57
Gender (M/F)	12/13	16/22	0.84
FEV_1_ (L)	2.8±0.8	3.3±0.7	0.02
FEV_1_ (%)	103.2±18.1	112.4±13.5	0.06
FVC (L)	3.7±0.9	3.9±0.9	0.31
FVC (%)	110.9±17.2	111.0±13.9	0.20
FEV_1_/FVC (%)	77.3±5.7	83.3±4.1	<0.001
Pack-years	27.1±14.6	0.0±0.0	<0.001
SpO_2_ (%)	96.6±1.4	96.7±1.4	0.79
BMI (Kg/m^2^)	23.9±4.1	26.4±3.5	0.01

M/F: male/female, FEV_1_: forced expiratory volume in the first second, FVC: forced vital capacity, SpO_2_: pulse oximetry, BMI: body mass index. The values are presented as the means and standard deviations. The statistical analysis was performed by t test or Chi-square.

We identified systemic inflammation with higher numbers of neutrophils (p=0.004) and higher serum levels of TNF-α (p<0.001) in active smokers when compared with never-smoker healthy controls (Table 2). We did not find differences in the secretion of TNF-α and IL-6 between the spontaneous PBM cultures of different groups.

**Table 2:** Peripheral blood cells, serum values and spontaneous monocyte secretion of TNF-α and IL-6

	**Smokers**	**Controls**	**p**
	**N=25**	**N=38**	
Leukocytes (cel/mm^3^)	7249.2±2071.7	6110.5±1111.8	0.09
Neutrophils (cel/mm^3^)	4406.7±1802.7	3392.2±796.6	0.004
Lymphocytes (cel/mm^3^)	1879.6±480.5	1885.8±512.9	0.96
TNF-α serum (pg/ml)	5.2 (4.3-5.8)	3.6 (3.4-3.9)	<0.001
TNF-α monocytes(pg/ml)*	34.6 (16.1-55.7)	57.4 (22.2-100.9)	0.09
IL-6 serum (pg/ml)	0.40 (0.20-0.80)	0.30 (0.2-0.4)	0.45
IL-6 monocytes (pg/ml) *	1392.6 (272.6-2277.7)	1006.3 (281.9-3048.9)	0.93

TNF-α: tumor necrosis factor alpha, IL-6: interleukin 6, TNF-α and IL-6 monocytes*: measurements of the supernatant of spontaneous peripheral monocyte cultures.

The values are presented as the means and standard deviations or as medians and quartiles. The statistical analysis was performed by t test or Mann–Whitney.

**Figure 1:**
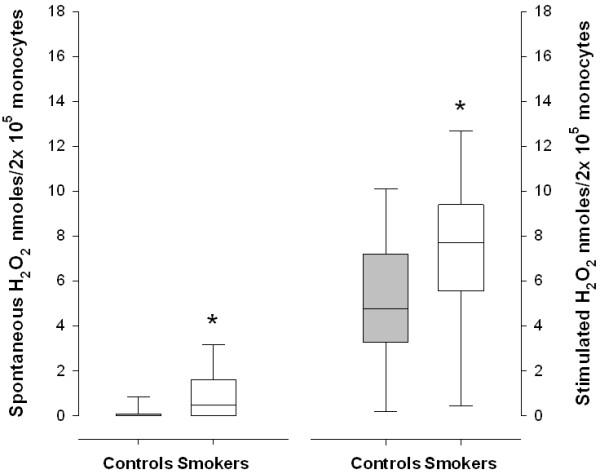
**The secretion of hydrogen peroxide, spontaneous and stimulated with phorbol myristate acetate, in smokers and healthy controls.** Medians (lines), interquartiles (boxes) and ranges (error bars) are shown. * p<0.001 when comparing controls and smokers.

The spontaneous and stimulated PBM culture secretions of H_2_O_2_ were statistically higher in the smokers than in the healthy controls (p<0.001) (Figure 1). The H_2_O_2_ secretion increased significantly after stimulation with PMA in both groups (p<0.001).

When the smokers and the controls were analyzed in the multiple regression analysis adjusted for age, gender, FEV_1_ (L) and BMI, there was a significant positive association between pack-years of smoking and the spontaneous secretion of H_2_O_2_ by PBM culture (Table 3). However, the stimulated PBM culture secretion of H_2_O_2_ was not associated with smoking pack-years after adjustments for potential confounding variable. Pack-years of smoking also exhibited a positive correlation with the number of leukocytes in the peripheral blood (coefficient=5.22, p<0.001) and the serum levels of TNF-α (coefficient=5.41, p=0.007).

**Table 3:** **Multiple regression models for spontaneous and stimulated monocyte production of H**_**2**_**O**_**2**_

**Model**	**Coefficient**	**Coefficient**
	**95% confidence interval**	**95% confidence interval**
	**Spontaneous production H**_**2**_**O**_**2**_	**Stimulated production H**_**2**_**O**_**2**_
Pack-years	0.03(0.01; 0.05)	0.04(−0.03; 0.10)
Pack-years	0.03(0.001; 0.05)	0.06(−0.02; 0.14)
TNF-α serum (pg/ml)	−0.19(−0.85; 0.48)	−0.48(−1.35; 0.39)
Pack-years	0.02(0.001; 0.04)	0.04(−0.03; 0.11)
TNF-α monocytes (pg/ml)	−0.0007(−0.002; 0.0007)	−0.001(−0.009; 0.004)
Pack-years	0.02(0.005; 0.06)	0.03(−0.03; 0.10)
IL-6 serum (pg/ml)	0.19(−0.40; 0.77)	0.61(−1.21; 2.43)
Pack-years	0.0(0.005; 0.05)	0.06(−0.006; 0.13)
IL-6 monocytes (pg/ml)	−1.5e-04(−3.7e-04; 6.5e-06)	−2.5e-04(−9.1e-04; 4.0e-04)
Pack-years	0.02(−0.004; 0.04)	0.01(−0.06; 0.09)
Neutrophils (cel/mm^3^)	0.0001(−0.00002; 0.0003)	0.0005(−0.0002; 0.001)

H_2_O_2_: peroxide hydrogen; TNF- α: tumor necrosis factor alpha; IL-6: interleukin 6. Monocyte production of H_2_O_2_ was stimulated by phorbol myristate acetate.

All of the multiple regression models with robust standard errors were adjusted for potential confounding variables (age, gender, forced expiratory volume in the first second and body mass index).

We did not find associations between H_2_O_2_ secretion with systemic inflammation evaluated by serum values and PBM culture secretion of TNF-α or IL-6 or the number of neutrophils in the peripheral blood (Table 3).

## Discussion

This study aimed to evaluate the relationship between systemic oxidative stress and intensity of smoking exposure. The main finding was that the smokers exhibited significantly higher secretion of H_2_O_2_ than the healthy controls and that there was a significantly positive association of oxidative stress with pack-years of smoking. These results confirm that smoking itself is an important determinant of oxidative stress and that a higher consumption of cigarettes is associated with higher levels of oxidative stress [12,14,20]. We also reinforce previous findings that smokers exhibit evidence of systemic inflammation compared with healthy controls [7,21].

The relationship between intensity of smoking exposure, through the evaluation of smoking pack-years, and oxidative stress has previously been investigated in the literature. However, our study is the first to show a significant association between pack-years of smoking and the secretion of H_2_O_2_ by PBMs. Thomassen et al. [22] did not find a correlation between the number of pack-years of smoking and the secretion of H_2_O_2_ by alveolar macrophages when assessing smokers and non-smokers. Similarly, Puri et al. [23] found no correlation between exhaled ethane air levels and cumulative smoking status. It is difficult to compare our results with those of these studies. The study by Thomassen et al. [22] used different cultures of cells to evaluate the secretion of H_2_O_2_. Those authors assessed alveolar macrophages, whereas we evaluated PBM cultures. Similarly, Puri et al. used a different outcome to quantify oxidative stress. However, the sample size of these studies was smaller than that in our study.

In this study, we demonstrated the higher secretion of H_2_O_2_ in smokers by PBM cultures, either spontaneously or after stimulation. Nowak et al. [2] demonstrated increased content of H_2_O_2_ in the expired breath condensate of cigarette smokers compared with controls. Our results are also in agreement with those of Ishida et al. [24], who identified higher H_2_O_2_ production in alveolar macrophage cultures of rats exposed to cigarette smoke. In contrast to our results, van Beurden et al. [14] did not detect differences in the release of superoxide dismutase by PBM cultures, either spontaneously or after stimulation with PMA, between COPD current/ex-smoker patients and healthy controls, and the smoking history did not influence the results when compared by covariance analysis between groups. The authors concluded that superoxide dismutase is only one of the oxidants that are produced by PBM culture; these cells also produce H_2_O_2_ and hydroxyl radicals. Therefore, the fact that the secretion of H_2_O_2_ by PBM culture is higher in smokers may indicate an imbalance in which the production of reactive oxygen species exceeds the capacity of the antioxidant defense systems in the systemic microenvironment. The oxidative stress may already be occurring to produce oxygen free radicals [25].

Our results showed higher secretion in H_2_O_2_ after stimulation with PMA in both groups, and the smokers exhibited higher production than the controls. This result indicates that the smokers included in our study still have monocytes with efficient immune capacity [26]. We can speculate that monocyte phagocytosis in smokers is still functional, although we did not evaluate the immune capacity in our study. In contrast, Correa et al. showed lower fungicidal activity and secretion of H_2_O_2_ by the monocytes of patients with peripheral atherosclerosis obliterans compared with those of control subjects.

Cigarette smoke induces the enhanced recruitment of mononuclear phagocytes and polymorphonuclear cells into the lower airways [2,12], and these cells modify oxygen metabolism and release additional H_2_O_2_ and other reactive oxygen species [25]. In our results, we identified a statistically higher number of neutrophil cells in the peripheral blood of smokers in comparison with that of healthy controls and a positive association between the numbers of leukocytes cells and pack-years of smoking. These observations are in accordance with a previous study that demonstrated an accelerated release of polymorphonuclear leukocytes from the bone marrow after smoking exposure [6].

The systemic inflammation induced by smoking includes neutrophilia and cytokines [6,27,28]. In our study, we found higher levels of TNF-α in smokers compared with healthy controls. Similarly, Tanni et al. [28] and Pretescu et al. [9] showed higher TNF-α levels in smokers compared with healthy non-smokers. We did not find differences in the cytokine secretion by the PBM culture in both groups, and the literature reveals controversial results that are dependent on the cell culture methods [7,21]. In agreement with our results, Ryder et al. [21] utilized cell cultures without stimulus and did not find increased values of TNF-α production by PBM cultures in smokers compared with controls. In contrast, Zeidel et al. [7] identified increased production of the pro-inflammatory cytokines (IL-1β, IL-6 and TNF-α) in smokers compared with non-smoking subjects, however, these authors analyzed cell cultures stimulated with lipopolysaccharide.

The limitations of our study need to be addressed. First, this study is cross sectional, and we are not explaining the cause-and-effect relationships among events. Second, we did not evaluate whether different smoking pack-years or cigarettes-day can induce greater secretion of H_2_O_2_ to confirm our results. Third, we did not assess the other biomarkers of oxidative stress and antioxidants to better understand the biological response to smoking pack-years.

In conclusion, we identified an association between higher secretions of H_2_O_2_ and smokers compared with healthy controls. The influence of pack-years smoking may be a key modifiable factor in oxidative stress associated to smoking.

## Abbreviations

BMI: Body mass index; COPD: Chronic obstructive pulmonary disease; FEV_1_: Forced expiratory volume in the first second; FVC: Forced vital capacity; H_2_O_2_: Hydrogen peroxide; IL: Interleukin; PBM: Peripheral blood monocyte; PMA: Phorbol myristate acetate; PMN: Polymorphonuclear leukocytes; RPMI: Roswell park memorial institute; TNF-α: Tumor necrosis factor alpha.

## Competing interests

None of the authors has any potential conflicts of interest.

## Authors' contributions

The authors’ responsibilities were as follow. SET: performed selection and the medical assessment of the individuals, statistical analysis and interpret the data and draft the final manuscript; CRC: laboratory analysis; AYOA: conducted the laboratory analysis; SAV: performed the medical assessment; LSC: performed the medical assessment, IG: had overall responsibility for the study, designed the research, analyzed and interpret the data, and wrote the final manuscript. All the authors contributed to the revision of the manuscript.

## Funding

Research Grant from FAPESP (Fundação de Amparo à Pesquisa do Estado de São Paulo, São Paulo, Brazil) N º 03/05285-1.
